# Bioactive Compounds and Functional Foods in Human Health—Bridging Mechanisms and Applications for Future Nutrition

**DOI:** 10.3390/nu17223586

**Published:** 2025-11-17

**Authors:** Vassilis Athanasiadis, Stavros I. Lalas

**Affiliations:** Department of Food Science and Nutrition, University of Thessaly, Terma N. Temponera Street, 43100 Karditsa, Greece; slalas@uth.gr

The role of bioactive compounds in the diet has moved from the periphery to the center of scientific and societal debate in both research and public health. Over the past decade, evidence has accumulated showing that polyphenols, carotenoids, and other bioactive molecules can influence inflammation, oxidative stress, and metabolic regulation. These findings are particularly important for chronic diseases such as cardiovascular disease and type 2 diabetes, where proper nutrition continues to act as an adjunct to medical therapy [1,2]. However, the benefits of these compounds are not guaranteed. Their efficacy depends on bioavailability, which is shaped by food structure and, increasingly, by interactions with the gut microbiota [1,2,3].

Despite this progress, many questions remain unanswered. We still lack a clear picture of how phytochemicals are absorbed and metabolized in real-world dietary contexts. Food matrices, preparation methods, and microbial interactions can alter outcomes in ways that are difficult to predict [2]. These uncertainties explain why clinical outcomes are sometimes inconsistent and highlight the need for more integrated approaches. Exploring these mechanisms is not only scientifically demanding, but also provides a foundation for translating laboratory data into realistic dietary practices [2,4].

The contributions to this Special Issue make important steps toward filling these gaps. Several publications examine how bioactive compounds interact with the microbiome [5], mitochondrial function [6], and lipid metabolism [7]. Together, they demonstrate a shift in the field—from isolated biochemical observations to a system-level understanding of nutrition. This approach is a necessary condition for the practical application of functional foods. A clear understanding of the mechanisms, especially biomolecular interactions, is essential for the reproducibility and clinical validity of the results [1,8].

Personalized nutrition is expected to be a central focus of future research and clinical activity. Genetic diversity, microbiome composition, and lifestyle factors all shape individual responses to nutrition. Although personalized nutrition is of particular interest, its implementation must be based on documented scientific and functional data [5,9]. Without a scientific basis, personalized nutrition may become a marketing fad rather than a meaningful therapeutic approach. Current research on nutrient–genotype interactions and microbiome-based nutritional strategies suggests that personalized recommendations could ultimately improve therapeutic outcomes [4,9,10].

The need for sustainable solutions makes it imperative to exploit alternative sources of bioactive compounds, such as neglected crops and agro-industrial by-products, placing nutritional research in the broader context of ecological responsibility. For example, olive leaves and citrus peels—by-products of the olive oil and juice industries, respectively—have emerged as promising sources of polyphenols with antioxidant and anti-inflammatory properties [11,12]. Similarly, underutilized crops such as amaranth and *Moringa oleifera* offer bioactive profiles that support both nutritional and ecological goals [13]. Advances in extraction and processing will be crucial to ensure that functional foods contribute not only to human health but also to ecological resilience [14]. In parallel, regulatory frameworks need to evolve, aiming to establish internationally harmonized standards, so that functional foods can gain consumer trust and be widely integrated into the market [9]. Looking ahead, several challenges remain in translating mechanistic insights into actionable dietary recommendations. Key questions include how to account for individual variability in microbiome composition, how to ensure reproducibility across diverse populations, and how to integrate emerging molecular data into practical guidelines. Addressing these issues will require coordinated efforts across nutrition science, clinical research, and regulatory policy [15,16].

In conclusion, bioactive compounds and functional foods have significant potential, the exploitation of which requires, beyond laboratory documentation, interdisciplinary collaboration and applied research. The transition from theory to practice requires interdisciplinary synergy and institutional alignment, with particular attention to environmental and regulatory parameters. If these elements are aligned, functional nutrition could become one of the defining public health strategies of the coming decades.
